# Left atrial function for outcome prediction in severe sepsis and septic shock: An echocardiographic study

**DOI:** 10.4103/0972-5229.56050

**Published:** 2009

**Authors:** Amr S. Omar, Masood ur Rahman, Said Abuhasna

**Affiliations:** **From:** Department of Critical Care Medicine, Tawam Hospital, AL Ain, UAE

**Keywords:** Left atrial function, mortality, septic shock

## Abstract

Left ventricular function and B-type natriuretic peptide (BNP) assessments are used to predict mortality in septic patients. Left atrial function has never been used to prognosticate outcome in septic patients.

**Objectives:**

To assess if deterioration of left atrial function in patients with severe sepsis and septic shock could predict mortality.

**Methods:**

We studied 30 patients with severe sepsis or septic shock with a mean age of 49.8±16.17. Echocardiographic parameters were measured on admission, Day 4, and Day 7, which comprised left ventricular ejection fraction (EF), and atrial function that is expressed as atrial ejection force (AEF). All patients were subjected to BNP assay as well. Multivariate analyses adjusted for APACHE II score was used for mortality prediction.

**Results:**

The underlying source for sepsis was lung in 10 patients (33%), blood in 7 patients (23.3%), abdomen in 7 patients (23.7%), and 3 patients (10%) had UTI as a cause of sepsis. Only one patient had CNS infection. In-hospital mortality was 23.3% (7 patients). Admission EF showed a significant difference between survivors and non survivors, 49.01±6.51 *vs..* 56.44±6.93% (*P*<0.01). On the other hand, admission AEF showed insignificant changes between the same groups, 10.9±2.81 *vs.* 9.41±2.4 k/dynes *P*=0.21, while BNP was significantly higher in the non survivors, 1123±236.08 *vs.* 592.7±347.1 pg/ml (*P*<0.001). The predicatable variables for mortality was Acute Physiology and Chronic Health Evaluation II score, BNP, then EF.

**Conclusion:**

In septic patients, left atrial function unlike the ventricular function and BNP levels can not be used as an independent predictor of mortality.

## Introduction

Predictors of outcome in critical care are well described and they include clinical, diagnostic, and physiologic variables[1] The acute physiology and chronic health evaluation II (APACHE II) scoring system is commonly used in the medical and surgical intensive care (MSICU) population to prognosticate outcome, and to compare acuity of medical care in different intensive care units.[2] Transthoracic echocardiography (TTE) is a widely recognized non invasive clinical tool in the assessment of patients with cardiovascular disease. The clinical impact of TTE on the daily bedside medical management decisions for patients in medical ICU has been previously described.[3–5] TTE diagnostic variables have been shown to be predictive of mortality in non-critical care cardiovascular patients with diastolic dysfunction.[6] But no direct relation had been made between atrial function and outcome of sepsis in various studies

Abnormalities of cardiac function are quite common in patients with sepsis. The prevalence of this transient phenomenon critically depends on the population studied, the definition applied, and the time point during the course of the disease. Approximately 50% of patients with severe sepsis and septic shock seem to have some form of impairment of left ventricular systolic function.[4]

The hemodynamic pattern in human septic shock is generally characterized by a hyperdynamic circulatory state including decreased systemic vascular resistance and a markedly increased cardiac index after adequate fluid resuscitation. Nevertheless, several studies have revealed clear evidence of intrinsically depressed left ventricular performance in patients with septic shock. The phenomena of myocardial depression in sepsis was first described by Parker, *et al*.[7]

## Aim of the study

The purpose of our study was to assess the utility of atrial function in predicting mortality in the ICU population with severe sepsis or septic shock. To our knowledge, no previous studies have addressed this issue. Our hypothesis was as the atrium share the same pathophysiological effects as the ventricles, assessment of the atrial function may be used as an alternative easy method of assessing the severity of myocardial dysfunction in sepsis and may therefore help to predict mortality.

## Materials and Methods

Following approval of the Tawam/Johns Hopkins Hospital Ethics Committee, we prospectively included 30 patients in the study from January 2007 to May 2008, with a mean age of 49.8±16.7 years. Inclusion criteria included patients with severe sepsis or septic shock. Patients with pre-existing heart failure or chronic renal failure were excluded.

All patients were subjected to have:

### Transthoracic echocardiography (TTE)

**Figure 1:**
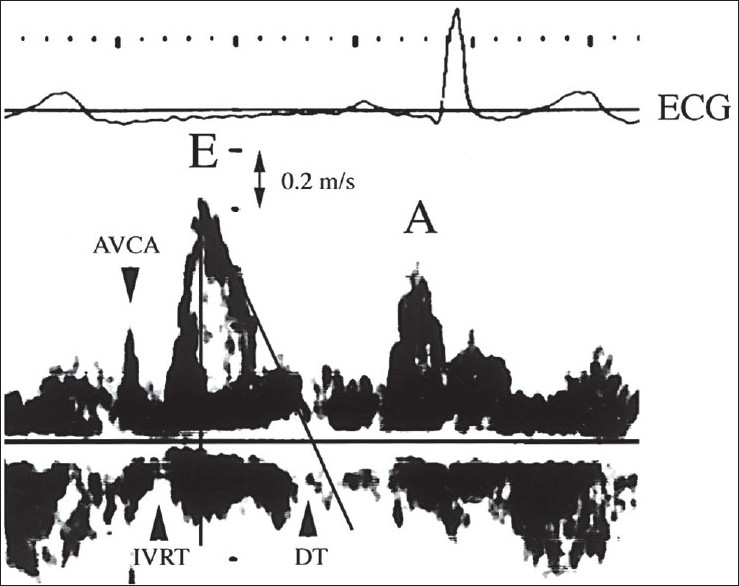
Transmitral left ventricular inflow velocity pattern obtained by pulsed Doppler echocardiography. Parameters derived are: E = Peak early diastolic left ventricular inflow velocity; A = left ventricular inflow velocity at atrial contraction; DT = deceleration (by extrapolation of E to the baseline); IVRT = isovolumetric relaxation time; AVCA = aortic valve closure artifact((31)).

The study was performed utilizing a General Electric Vivid *i* Sonos with a 2.5-MHz transducer. Two-dimensional and pulsed Doppler echocardiograms were obtained at rest with the patient placed in the left lateral position to evaluate left ventricular size and left ventricular systolic function. Echo parameters measured included the following dimensions 1) left ventricular end diastolic diameter (LVEDD) and left ventricular end systolic diameter (LVESD), 2) left atrial dimensions (length, diameter, and volume by planimetry), and 3) mitral orifice by planimetry The following functions were calculated 1) systolic left ventricular function namely the ejection fraction (EF%) and 2) Left atrial function after cardioversion expressed as · Atrial ejection force (AEF) =0.5×*P*×Mitral orifice area×(Peak A velocity)^2^. The unit of force would thus be measured in g-cm/s^−2^ or dynes while *P*=product of density of the blood (*P*=1.06g/cm^3^).[8] Measures were repeated on the 4^th^ and 7^th^ days of admission [Figure 1].

### BNP Measurements

Plasma B-type natriuertic peptide (BNP) concentrations were measured as previously described using the Triage BNP meter (Biosite Diagnostics, San Diego, CA).[9] The first BNP sample was taken on admission to the ICU (Day 1). BNP levels were determined for each patient on admission, Day 4, and Day 7.

### Other Data Collection

Baseline clinical variables including age, gender, cause of sepsis, and the admission APACHE II score were collected.[2] Other data collected included the requirements for mechanical ventilation (ventilation hours) and vasopressors, the length of stay in the ICU (LOS_ICU_) and in the hospital (LOS_HOS_), and the patient's outcome (alive or dead). Two patients developed atrial fibrillation, one patient reverted chemically with IV amiodaron and the other patient developed it later in the course before he died.

Sepsis has been defined as the presence of the systemic inflammatory response syndrome (SIRS) in response to a culture-proven infection.[10] However, SIRS can result not only from infection, but also from a variety of conditions such as autoimmune disorders, vasculitis, thromboembolism, and burns or after surgery. The severity of sepsis is graded according to the associated organ dysfunction and hemodynamic compromise. The original definitions have been revisited by a group of experts,[11] but apart from expanding the list of signs and symptoms of sepsis, no relevant changes have been made. In a recently published review, Annane, *et al.*[12] proposed a very practical modification of the definitions including exact hemodynamic definitions of septic shock. It is important to recognize that the original definitions relied only on the degree of vasodilatation, whereas in the modification by both the International Sepsis Definition Conference[11] and Annane *et al.,*[12] myocardial depression defined as low cardiac index or echocardiographic evidence of cardiac dysfunction has been included in the definition of severe sepsis [Table 1].

**Table 1:** Definitions of SIRS and different degrees of severity of sepsis[11,13]

Condition	Description
SIRS	Two or more of the following conditions: temperature > 38.5°C or < 35.0°C; heart rate of >90 beats/min; respiratory rate of >20 breaths/min or PaCo2 of <32 mm Hg; and WBC count of >12,000 cells/mL, <4,000 cells/mL, or > 10% immature (band) forms
Sepsis	SIRS in response to documented infection (culture or Gram stain of blood, sputum, urine, or normally sterile body fluid positive for pathogenic microorganism; or focus of infection identified by visual inspection, e.g., ruptured bowel with free air or bowel contents found in abdomen at surgery, wound with purulent discharge)
Severe sepsis	Sepsis and at least one of the following signs of organ hypoperfusion or organ dysfunction: areas of mottled skin; capillary refilling of > 3 s; urinary output of < 0.5 mL/kg for at least 1 h or renal replacement therapy; lactate > 2 mmol/L; abrupt change in mental status or abnormal EEG findings; platelet count of < 100,000 cells/mL or disseminated intravascular coagulation; acute lung injury/ARDS; and cardiac dysfunction (echocardiography)
Septic shock	Severe sepsis and one of the following conditions: systemic mean BP of <60 mm Hg (<80 mm Hg if previous hypertension) after 20–30 mL/kg starch or 40–60 mL/kg serum saline solution, or Pulmonary capillary wedge pressure (PCWP) between 12 and 20 mm Hg; and need for dopamine of > 5 *μ*g/kg/min, or norepinephrine or epinephrine of > 0.25 *μ*g/kg/min to maintain mean BP at > 60 mm Hg (80 mm Hg if previous hypertension). Refractory septic shock - need for dopamine at > 15 *μ*g/kg/min, or norepinephrine or epinephrine at 0.25 *μ*g/kg/min to maintain mean BP at > 60 mm Hg (80 mm Hg if previous hypertension)

### Statistical analysis

The description of the data were done in the form of mean ± SD for quantitative data and frequency and proportion for qualitative data. The analysis of the data was done to test the statistically significant difference between groups. For quantitative data, a student's t test was used to compare the two groups. For qualitative data, a Chi-square test was used and Odds Ratio was detected. Multivarate regression analysis was done for significant data in an univarate analysis.[13] The primary outcome for the study was defined as ICU mortality. Clinical and echocardiographic data were entered into a database (Microsoft Excel 97, Redmond, WA, USA) and statistical analyses were performed (SPSS Inc., Version 10.0.7 Chicago IL, USA).

N.B: P is significant if ≤ 0.05 at a confidence interval of 95%.

## Results

The patients' baseline characteristics are presented in Table 2. All 30 patients stayed in the ICU for more than 48 hours; 18 males and 12 females were included in the study. The underlying cause of sepsis was pneumonia in ten patients (33%), blood stream infection in seven patients (23.3%), intrabdominal sepsis in seven patients (23.7%), and three patients (10%) had urinary tract infection (UTI) as a cause of sepsis, while only one patient had a central nervous system (CNS) infection. A total of 20 patients were admitted with a diagnosis of severe sepsis and ten patients were admitted with a diagnosis of septic shock. A total of 20 patients required norepinephrine and two patients also received vasopressin at admission. Seven other patients required norepinephrine, of those five patients also received vasopressin at some stage in their ICU stay. The in-hospital mortality was 23.3% (seven patients).

**Table 2:** Baseline patient characteristics

Total no. of patients	30
Male/Female ratio	18/12
Age, yrs	49±16.17
Septic shock/Sever sepsis ratio	20/10
Source of infection, n (%)	Pneumonia 10(33.3%)
	Blood stream infection 7(23.3%)
	Intra-abdominal sepsis 7 (23.3%)
	Urinary tract infection 3(10%)
	CNS infection 1(3.3%)
	Unidentified 2 (6.6)
Mechanical ventilation No. (%)	19
Mean ventilation hour, hrs	151.2±91.2
LOS_ICU_, days	8.2±5.1
LOS_HOS_, days	15.3±11.6
Mortality, n (%)	7(23.3%)
Admission BNP, pg/ml	716±39
APACHE II score	15.3±2.9
EF%	55±%

Table 3 shows that there were no statistical differences between septic shock and severe sepsis groups regarding the baseline ejection fraction (EF), atrial ejection force (AEF), length of stay in the hospital (LOS,^HOS^) and length of stay in the ICU (LOS^ICU^). The in-hospital mortality rates were significantly higher in the septic shock group.

**Table 3:** Comparison between the septic shock and severe sepsis groups

	Severe sepsis	Septic shock	P-value
EF	52.1±8.67	56.±6.5	0.17
AEF	10.6±2.54	10.6±2.9	0.9
LOS_HOS_	17.9±13.4	12.8±10.3	0.26
LOS_ICU_	9.2±5.32	7.2±4.8	0.3
Mortality	4/20 (20%)	3/10 (30%)	0.05

The APACHE II score was significantly higher in the group of non survivors (*P*-value =0.007).

## Echocardiographic changes

### Left ventricular function

**Figure 2:**
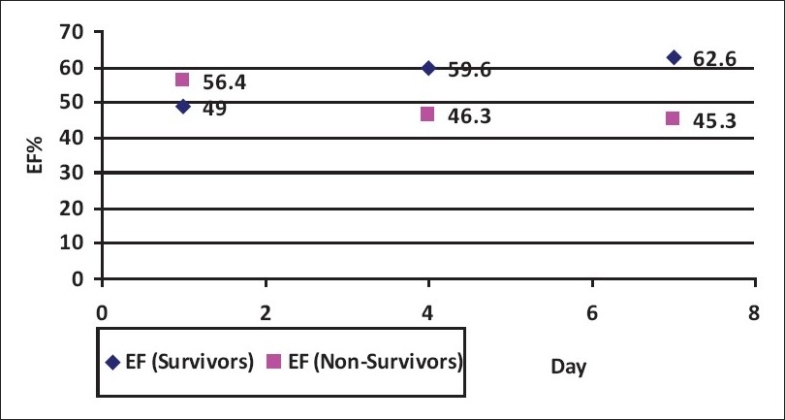
Admission and daily changes in EF in survivors and non survivors

The LV systolic function on admission was significantly higher in the group of non survivors (*P*-value = 0.018). The LVEDD did not show similar changes, and there was a significant difference in the LOS_ICU_ and LOS_HOS_ between the survivors and non survivors (*P*-value = 0.0001 and 0.001, respectively). However, daily changes in EF were noted in [Figure 2]; EF in survivors improved and remained significantly higher in the group of survivors as compared with the group of non survivors [Table 4].

**Table 4:** Comparison between the survivors and the nonsurvivors groups

	Survivors	Non-Survivors	*P* Value
No.	23	7	
Age, yrs	48.3±16.8	54.6±13.7	0.354
APACHE II	14.6±2.75	17.9±1.95	0.007
LOS_ICU_ days	9.5±4.51	2.4±1.27	0.00001
LOS_HOS_ days	18.0±10.8	2.9±1.46	0.00001
Baseline BNP	592.7±347.1	1123±236.1	0.001
Baseline AEF	10.9±2.81	9.4±2.4	0.21
LVEDD	5.8±0.5	4.0±0.4	0.179
LVESD	4.01±0.4	4.5±0.38	0.008
Baseline EF%	49.00±6.5	56.4±6.9	0.018

APACHE II: acute physiology and chronic health evaluation score); BNP: B-type natriuretic peptide; AEF: atrial ejection force; LVEDD: left ventricular end diastolic diameter; LVESD: left ventricular end systolic diameter; EF: ejection fraction

### Atrial function

**Figure 3:**
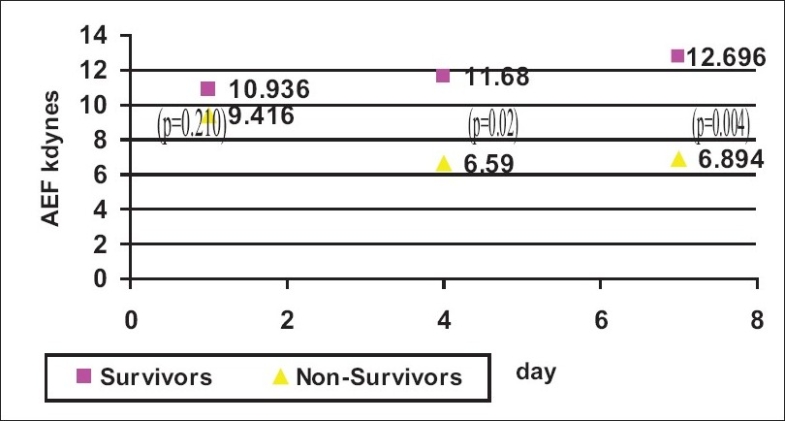
Admission and daily changes in AEF in survivors and non survivors

Admission AEF was higher in the group of survivors (Figure 3; *P*-value =0.21), however, subsequent daily AEF showed significant deterioration in the group of non survivors as compared with survivors (*P*-value = 0.02 and 0.004 on Day 4 and Day 7, respectively).

### BNP

Admission BNP concentrations were elevated in the studied group 716 ±39 pg/ml and BNP concentrations were significantly higher in the group of non survivors (*p*-value <0.0001). BNP concentrations remained significantly higher on the 4^th^ and 7^th^ days (*P*-value = 0.01 and 0.001, respectively in the group of non survivors).

### Mortality Prediction

By doing a multivariate logestic regression, the predicatable variables are APACHE II score, BNP, andEF.

## Discussion

The main objective of this study was to determine if echocardiographic data would add prognostication to existing clinical variables in ICU patients to analyze whether atrial ejection force (AEF) represents a sensitive and reliable parameter for the detection of cardiac alteration particularly in patients with severe sepsis and septic shock.

The findings of this study can be summarized as follows: 1) admission AEF did not differ between the survivors and the non surviviors, 2) admission APCHE II scores, EF, and BNP showed significant changes between survivors and non survivors, 3) AEF could not be used as an independent mortality preictor in severe sepsis and septic shock, and 4) AEF in survivors later in the course of sepsis tends to improve.

### Cardiac function

The phenomenon of sepsis-related cardiomyopathy has been described in many trials.[7,14] Parker, *et al.,*[15] were the first to describe left ventricular hypokinesis in septic shock in which patients with severe LVEF with an adequate LV stroke output could be maintained through acute LV dilatation.[16]

Our data showed that the admission LV systolic function was significantly higher in the group of non survivors (*P*-value=0.018). Daily changes in the EF were noted in [Figure 2]. EF tended to improve and remained significantly higher in the group of survivors. Improvement in contractility may result from the resolution of sepsis-induced cardiomyopathy.

Our data was supported by the study of Charpentier, *et al.,.*[4] who evaluated the cardiac performance in patients with sepsis by echocardiography and found an LVEF (using TTE) or a left ventricular fractional area contraction (LVFAC) using TEE of <50% in approximately 50% of patients with severe sepsis and septic shock. However, the typical pattern of left ventricular dilation in combination with an impaired LVEF was found in only one study by Ver Elst,*et al,,*[17] whereas in the study conducted by Charpentier, *et al.,*[4] ventricular dimensions were normal despite low LVEF. In a study by McLean, *et al.,*[18] seven patients (18% of the cohort) displayed Reversible Cardiac Dysfunction (RCD), which was characterized by an initially reduced LVEF (<55%) with subsequent normalization of LVEF (i.e., LVEF >55%).

### Atrial function

Determination of left atrial systolic function by measuring atrial ejection force (AEF) at left ventricular relaxation enables us to assess atrial contribution to the filling of the left ventricle.[10] Thus, AEF serves equally as an indirect parameter of left ventricular diastolic function (i.e., to assess left ventricular relaxation).

With regard to atrial function, we found that admission AEF was slightly higher (non significant) in the group of survivors (Figure 2: *P*-value = 0.210). Our hypothesis was that serial AEF may predict survival in patients with septic shock.

### BNP changes

The cardiac ventricles are the main source of circulating BNP in humans. The stimulus for BNP release is the stretching of the ventricular wall as a result of either volume expansion or pressure overload.[19] BNP levels are elevated in patients with symptomatic left ventricular dysfunction and correlate with filling pressures.[20]

Admission BNP concentrations were elevated in the studied group at 716 ±393 pg/ml and BNP concentrations were significantly higher in the group of non survivors (*P*-value <0.0001). BNP concentrations remained significantly higher on the 4^th^ and 7^th^ days (*P*-value = 0.01 and 0.001, respectively) in the group of non survivors [Figure 4].

BNP concentrations were increased in patients with severe sepsis or septic shock regardless of the presence or absence of cardiac dysfunction.[18]

### Mortality Prediction

Despite initial recovery from critical illness requiring intensive care unit (ICU) admission, many patients remain at risk of subsequent deterioration and death. This may result in readmission to the ICU or death in another ward or during the ICU readmission. Early identification of patients at the highest risk would allow resources to be targeted appropriately and prevent avoidable morbidity and mortality. ICU readmission rates have been advocated as a marker of ICU quality on the basis that early readmissions (within 48 hours) may indicate premature discharge or discharge to an inappropriate clinical area.[21,22]

The APACHE prognostic scoring system is a powerful predictor of hospital mortality in the medical and surgical intensive care unit (MSICU) patient population both in North America[23,24] and internationally.[25–28] The APACHE II scoring system is commonly used in the MSICU population to prognosticate outcome and to compare acuity of medical care in different intensive care units.[22]

Our data was consistent with the predictive value of the APACHE II score for mortality; the APACHE II score was significantly higher in the group of non survivors (*P*-value = 0.007).

In the landmark study by Parker, *et al*.,[7] patients were grouped according to their mortality, and patients showing left ventricular dilation and depression of LVEF had a good prognosis. Paradoxically, many studies using echocardiography showed that an impaired LVEF is associated with a poor prognosis.[4,16] This might be explained by the fact that in patients with septic shock, the measurement of LVEF alone does not sufficiently characterize the underlying hemodynamic pattern, and that outcome depends on parameters other than LVEF.

In a recent study done by Ritter, *et al.,*[29] the authors concluded that cardiac index and cardiac function index (CFI) both provide prognostic information for patients with severe sepsis or septic shock. In another study by Sawchuk, *et al.,*[30] the authors mention that TTE does not improve the prediction of outcome over APACHE II in medical-surgical intensive care.

Systolic myocardial dysfunction is present in 44% of the patients with severe sepsis or septic shock. In this setting, brain natriuretic peptide seems useful to detect myocardial dysfunction, and high plasma levels appear to be associated with a poor outcome of sepsis, but further studies are needed.[4]

BNP provided prognostic value for in-hospital mortality and length of stay in this mixed group of patients, which included patients with chronic cardiac dysfunction.[17]

## Conclusion

In septic patients, atrial function unlike ventricular function and BNP levels could not be used as an independent predictor of mortality.

This study had several limitations. First, we tried to match the recruitment time to the time of the onset of sepsis. However, as discussed previously this is nearly an impossible task mainly due to delays in the presentation to the ICU, the presentation of symptoms, and/or the reporting of the microbiology test results. Second, the relatively small sample size may reduce the power of some analyses (comparisons). Finally, the interpretations of cardiac function might be affected by the use of α-agonists such as norepinephrine. The use of inotropes in these patients might improve their cardiac function and lead to an overestimation of cardiac variables such as LVEF.
